# Reduction of the natural Activated protein C pathway activity significantly prevents coagulopathy in a murine model of acute traumatic coagulopathy

**DOI:** 10.1186/1757-7241-22-S1-O6

**Published:** 2014-07-07

**Authors:** Maria Guerreiro, Jordi L Tremoleda, Dan Frith, Chris Thiemermann, Karim Brohi

**Affiliations:** 1 Bart’s and the London school of Medicine and Dentistry, Queen Mary, University of London, UK; 2 William Harvey Research Institute, London, UK

## Background

Acute traumatic coagulopathy (ATC) identified in the first hour of trauma patients is associated with worse outcomes and increased risk of death [1]. Identification of critical coagulation pathways involved in ATC is important for future targeted drug discovery. Previous studies have demonstrated an activation of the thrombomodulin-protein C pathway during ATC [2]. We examined the effect of reduced thrombomodulin generation in an experimental model of ATC.

## Methods

Both wild type (WT) and modified Knock-in TM pro/pro mice (TMKI) were subjected to trauma (laparotomy with muscle injury and bilateral tibia-fibula fracture) and haemorrhage (40± 5% of estimated blood volume) to a target mean arterial blood pressure of 30 ± 5 mmHg. This model was developed to elicit coagulopathy as a response to trauma and bleeding thus mimicking the coagulopathy present in the injured patient. The animals were kept euthermic and were not resuscitated. After 60 minutes blood samples were taken and analysed via thrombelastometry tests.

## Results

All animals were shocked when compared with controls (lactate ≥ 7 mg/dL and ≤ 3 mg/dL; p<0.001). After trauma and haemorrhagic shock (THS), WT mice developed a significant coagulopathy, as measured by a reduction in clotting amplitude at 5 minutes (CA5’) (THS time 0 vs time 60 = 46.7± 1.37 vs 27.6±2.57 mm, p<0.0002) and in maximal clot firmness (MCF) (THS t0 vs t60 =62.7±1.1 mm vs 46± 3.3 mm, p<0.001). In the TMKI group, which entails a 1000 fold reduction in aPC production there was a correction of the coagulopathy (CA 5’: TMKI time 0 vs time 60 = 42.6± 2.3 vs 41.6±1.5 mm, p<0.0002 and MCF (TMKI t0 vs t60 =56.8±2 mm vs 55.1± 1 mm, p<0.001.

## Conclusion

Severe trauma shock induced a significant coagulopathy characterised by the generation of fragile clots. The reduction in activated protein C generation encompassed an improvement in the experimental coagulopathy therefore the protein C pathway seems to play an important role in acute traumatic coagulopathy.
